# Social Sensing of Heatwaves

**DOI:** 10.3390/s21113717

**Published:** 2021-05-26

**Authors:** James C. Young, Rudy Arthur, Michelle Spruce, Hywel T. P. Williams

**Affiliations:** 1 Computer Science, Innovation Centre, University of Exeter, North Park Road, Exeter EX4 4RN, UK; r.arthur@exeter.ac.uk (R.A.); ms886@exeter.ac.uk (M.S.); h.t.p.williams@exeter.ac.uk (H.T.P.W.); 2 Alan Turing Institute, 96 Euston Road, London NW1 2DB, UK

**Keywords:** heatwave, heat, extreme weather, natural hazards, social sensing, social media

## Abstract

Heatwaves cause thousands of deaths every year, yet the social impacts of heat are poorly measured. Temperature alone is not sufficient to measure impacts and “heatwaves” are defined differently in different cities/countries. This study used data from the microblogging platform Twitter to detect different scales of response and varying attitudes to heatwaves within the United Kingdom (UK), the United States of America (US) and Australia. At the country scale, the volume of heat-related Twitter activity increased exponentially as temperature increased. The initial social reaction differed between countries, with a larger response to heatwaves elicited from the UK than from Australia, despite the comparatively milder conditions in the UK. Language analysis reveals that the UK user population typically responds with concern for individual wellbeing and discomfort, whereas Australian and US users typically focus on the environmental consequences. At the city scale, differing responses are seen in London, Sydney and New York on governmentally defined heatwave days; sentiment changes predictably in London and New York over a 24-h period, while sentiment is more constant in Sydney. This study shows that social media data can provide robust observations of public response to heat, suggesting that social sensing of heatwaves might be useful for preparedness and mitigation.

## 1. Introduction

Heatwaves are predicted to increase in frequency, duration and intensity as a result of climate change [1]. The formation of heatwaves can vary based on location, yet is fundamentally a result of high pressure in the upper atmosphere trapping hot, low-pressure air close to the Earth’s surface for extended periods [2]. The impacts of heatwaves are wide-ranging, affecting human health (e.g., an estimated 70,000 additional deaths were caused by the 2003 European heatwave [3]), the natural environment (e.g., the 15 million hectare Russian forest fire during the 2010 heatwave [4]) and economic activity (e.g., economic losses of $2.4 trillion a year by 2030 are projected due to heat stress [5]). The impacts of heat on wellbeing are equally as significant. Liu et al. [6] found a significant increase of mental illness related hospital admissions as a result of heatwaves. Additionally, a meta-analysis conducted by Gao et al. [7] found that each 1 ${}^{\circ }$C increase in temperature was significantly associated with a 1% increase in suicide incidence.

Whilst heatwaves can be severe, they are experienced differently depending on local climate and preparedness. Heatwaves are often defined locally relative to regional conditions [8]. Xu et al. [9] compared universal definitions to different locations, finding that definitions that were city- or region-specific were more likely to be useful. This variation and subjectivity means that global efforts to mitigate the effects of heatwaves are also varied, with heatwaves seen as a threat in some countries but not in others.

Differing local perceptions of heatwaves are exemplified by the United Kingdom, the United States of America, and Australia, which have all experienced serious heatwaves in the 21st century [10]. The average minimum/maximum temperatures experienced in these countries differ considerably: 6.0 ${}^{\circ }$C/12.9 ${}^{\circ }$C in the UK, 9.7 ${}^{\circ }$C/17.2 ${}^{\circ }$C in the US, and 11.8 ${}^{\circ }$C/23.6 ${}^{\circ }$C in Australia [11]. The UK’s national weather service, the Met Office, defines a heatwave as three consecutive days where a region’s average daily maximum temperature (ADMT) is above its 90th percentile (i.e., in London, 3 days over 28 ${}^{\circ }$C is a heatwave) [8]. Public perception of heatwaves is generally positive in the UK; Bruine de Bruin et al. [12] showed that exceptionally hot UK summers were usually considered as positive weather events, despite the potential severity of heatwaves. In the US, the National Oceanic and Atmospheric Administration (NOAA) define heatwaves as a “2 or more day period of unusually hot weather” [2]. However, local variations exist; for instance, in New York, three days over 90 ${}^{\circ }$F (32 ${}^{\circ }$C) is classified as a heatwave [13]. Despite extreme heat causing more deaths in the US than any other weather event, the US public’s perceived risk of heat varies greatly [14,15]. Notably, those living in colder states were least aware of the risks associated with heat, despite being at most risk due to their lack of acclimatisation and awareness. The same heatwave underestimation was found with the elderly and vulnerable, two demographics that are disproportionately impacted by heat [16]. Meanwhile, Australia experiences frequent heatwaves, with nine of its ten hottest years recorded having occurred since 2005 [17]. The heatwave classification used by the Australian national weather service, the Bureau of Meteorology (BOM), is called the Excess Heat Factor (EHF) [18], which calculates heatwave intensity by considering anomalous short- and long-term daily mean temperatures, accounting for acclimatisation. A study into the perceptions of Adelaide residents regarding heat showed that most respondents followed heatwave news closely and that it was of significant importance to them [19]. Additionally, there was greater expressed concern for societal heatwave impacts than personal heatwave impacts.

Moving beyond between-country comparisons, it also appears that heatwaves may be experienced differently within countries. Widespread evidence for ‘urban heat island’ effects, which make cities warmer than surrounding rural areas, suggests that cities may be most at risk from heatwaves [20,21]. This is supported by research showing that heatwaves further enhance the urban-rural heat disparity [22]. Dong et al. [23] investigated the risk this posed to human health, finding that during a heatwave in the city of Wuhan in China, the health risk close to the city centre was 1.6 times greater than the surrounding rural areas.

Whilst the physical mechanisms of heatwaves are relatively well understood by meteorologists, the social impacts remain poorly observed. The lack of understanding of social impacts hinders the adoption of impact-based forecasting approaches and reduces the ability to mitigate the effects of heat [24]. Despite being of clear value, obtaining relevant impact data is often slow and costly, with common sources including news reports, citizen data and insurance data. One approach that has the potential for addressing this outstanding lack of social impact data is social sensing. Social sensing is the utilisation of humans as sensors, extracting data from social media platforms to enable insights into real-world systems. Social sensing has many social applications, including political modelling [25] and crime sensing [26]. Recently, the use of social sensing to understand natural hazards has been gaining traction, improving the prediction, detection, and characterisation of hazard events. An early example is that of Sakaki et al. [27], who implemented a Twitter-based system able to classify and locate 96% of earthquakes in Japan above a given threshold. Social sensing of extreme weather events has had successes in the detection of UK floods [28], measurement of storm impacts [29] and the location of wildfires [30]. Whilst Facebook and Instagram have been used for social sensing with varying success [31,32,33,34], the more reactive nature of Twitter is better suited for social sensing, with news received faster through Twitter than through traditional media [35]. Twitter’s high popularity further justifies it as a suitable platform for social sensing, with 22%, 23% and 21% of the population in the US, UK and Australia using it, respectively [36,37,38]. Additionally, Twitter’s public API and openness for data collection [39] make it a more scalable solution for providing impact data for assisting impact-based forecasting, and thus it was chosen for this study.

The aim of this study is to use social sensing to explore the impacts of heatwaves on human populations. A comparison of three countries (UK, US and Australia) will be used to show differences in perception of heatwaves that may arise from local climate and expected temperatures. The volume of social media activity and sentiment analysis will be used as two indicators for the social impacts of heat. Effects will be explored for the three countries as a whole, as well as at the city scale, focusing on the largest city in each country (London, New York and Sydney [40]). The aim is to demonstrate that social sensing can be effectively used to understand the social impacts of heat at different scales, as well as to illustrate the differing perceptions of heatwaves in different regions. This will expand the evidence base and contribute to better management of heatwaves and their impacts.

The structure of this paper is as follows: Section 2 provides an overview of the data sources used, including data characteristics and processing methods. Section 3 presents the results. Section 4, Section 5 and Section 6 discuss the results and summarise limitations and opportunities for future work.

## 2. Methods

With the frequency of heatwaves increasing [1], the three significant heatwave events chosen for research all occurred in recent years, with a summary of them shown in Table 1. The UK heatwave period investigated for this paper is July–September 2018. As reported by McCarthy et al. [41], this was one of the warmest recorded UK summers, with temperature peaks coinciding with sharp spikes in daily death count and extensive wildfires. The heatwave period observed for the US was also July–September 2018. This was the fourth hottest summer in US history, with numerous state temperature records and particularly anomalous overnight temperature highs [42]. The Australian heatwave period selected was between December–February 2018/2019, and was the hottest summer on record by 0.86 ${}^{\circ }$C [43]. The unusual length, intensity and national coverage of the Australian heatwaves justify 2018/2019 as an appropriate summer for investigation.

### 2.1. Data Collection

#### 2.1.1. Twitter Data

Twitter is a social media microblogging platform where users produce character-restricted messages called tweets. With 500 million daily tweets [44] and easy data availability via an open application programming interface (API) [39], Twitter is a popular platform for social sensing. Twitter data used in this work was collected via the Streaming API, retrieving tweets that contained the English-language keywords ‘heatwave’ or ‘drought’. Drought tweets were removed from the dataset after preprocessing due to the study’s focus on heatwaves, with the social response between the two being more diverse than initially anticipated.

Tweets are stored in a lightweight, versatile, human-readable format called JavaScript Object Notation (JSON) files [45]. Information regarding tweet metadata can be found at https://developer.twitter.com/en/docs/twitter-api (accessed on 20 October 2020). There has been no manipulation or distribution of any data as per Twitter’s developer agreement [46].

Two datasets were collated for this work. Dataset 1 covers the 2018 European and US heatwave with 1,953,570 tweets created between 1 June 2018–31 August 2018. Dataset 2 covers the Australian heatwave of 2018/2019, with 763,022 tweets created between 1 December 2018–28 February 2019. Both datasets are continuous in time with no drop-offs or collection outages. Since these are keyword-based collections, the tweets collected can originate from anywhere on Earth, though in practice, most tweets originate from densely populated areas with high Twitter usage [47]. Restriction to the countries/cities of interest is performed using location inference (see below).

#### 2.1.2. Temperature Data

Temperature data for this project were collected from two sources: the UK Met Office for records covering the UK, and the US National Oceanic and Atmospheric Administration (NOAA) for records covering the US and Australia. The Met Office data was taken from the ‘Integrated Data Archive System’ (MIDAS) [48] and contained land surface temperature summaries. The NOAA data was taken from the ‘Global Surface Summary of the Day’ (GSOD) dataset [49]. Both data sources measured land surface temperature and are thus comparable.

### 2.2. Preprocessing

#### 2.2.1. Twitter Data

**Figure 1:**
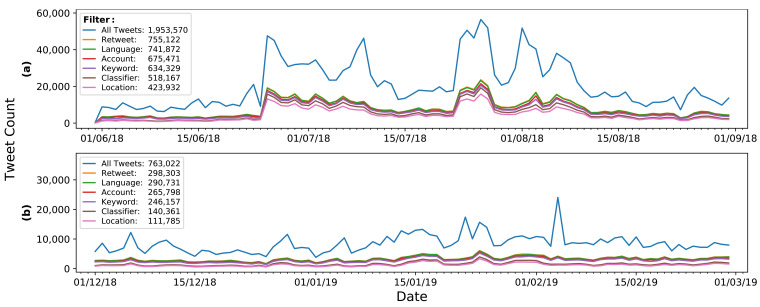
Change in tweet count at different stages of filtering for Dataset 1 (**a**) and Dataset 2 (**b**).

Manual inspection of a sample of 200 randomly chosen tweets from Dataset 1 indicated that approximately 28% of tweets were relevant to the study of heatwaves. The remainder included a variety of other non-relevant uses of the collection keyword such as the Martha and the Vandellas song "Heatwave". Therefore, a rigorous filtering process was implemented on Dataset 1, with the final pipeline being applied to Dataset 2 (discussed later). A summary of the steps taken is given below. Figure 1a shows the remaining daily tweet counts over the June–August 2018 period after filtering. Filtering steps were applied sequentially so that each filter operated on the tweets remaining after the previous filter had been applied.

##### Filter 1: Retweets and Quotes

This study is interested in unique responses to weather conditions, not endorsements or comments. Though the number of retweets or replies to a tweet could be correlated with relevance, it is also strongly influenced by the popularity of the user who made it [50]. To avoid confounding by social network effects all retweets have been removed. Replies have been excluded in the case when the respondent did not use the collection keywords in their message. Removing retweets and ambiguous replies caused the largest data reduction in the preprocessing, decreasing the dataset by 61%.

##### Filter 2: English Language

Since English-language keywords were used to collect the data, the datasets studied were 97% English-language tweets and therefore other languages were removed. A further 1% had an undefined language. These were classified using the language identification package ‘langdetect’ [51], with an additional 7756 English tweets detected.

##### Filter 3: Bot, News and Weather Accounts

‘Bot’ is an internet abbreviation for a robot, used as a label for internet activity that has not been directly produced by a human. As this is a social sensing project, removing bots ensured that the data corresponded to the human impacts of heat. Usually, a clear indication of a bot account is excessive tweeting. An effective rule of thumb [28] is to identify any user whose tweets account for more than 1% of the total volume of tweets collected. Whilst no accounts satisfied this condition, human accounts would often tweet a message identical to other users, usually to be entered into competitions. Therefore, all messages tweeted by separate users over four times were excluded.

Through manual observation, it was clear that the majority of the most active user accounts were weather stations or news accounts. Tweets from these users frequently contained automated weather recordings, with no human observations present. By inspecting the 100 highest tweeting accounts, a list of key terms found in these unwanted usernames was created (Table A1). If any username contained a word from this list, it was removed. This identified 11,820 unwanted accounts, with a random 1% account sample showing 95% were correctly classified. Upon removing duplicate tweets, and tweets from weather and news accounts, the tweet count decreased by 9%.

##### Filter 4: Term-Based Relevance

A random sample of 2727 remaining tweets was extracted and manually classified as relevant or irrelevant. A tweet was deemed relevant if it was not promoting or selling a product (i.e., ice-cream or swimming pools) and if it conveyed an attitude/consequence/ response to a current or recent heatwave. There was a 61.5% relevance at this stage, with the manual filtering highlighting 5 common topic categories in irrelevant tweets: K-Pop (Korean pop music), Western pop music, sports, sexual and other. Common words within each irrelevant topic were then compiled into a ‘black-list’ of terms, and if a tweet contained any of these words, it was omitted from the dataset. A full list of the words in each category that were used to exclude irrelevant tweets is shown in Table A2. Application of this filter reduced the remaining dataset by 7%.

##### Filter 5: Machine Learning Relevance Classifier

The tweet sample taken in the previous step indicated a high proportion of irrelevant tweets (38.5%), however as the term-based relevance filter removed only 6% of tweets, a machine learning approach was taken to create a second relevance filter. A training set was created by filtering a random sample of 2557 tweets from the filtered dataset. These tweets were manually classified as either relevant or irrelevant using the same criteria as Filter 4.

Three classification models were evaluated: Multinomial Naïve Bayes, Support Vector Machine, and Logistic Regression. These models were selected due to their success in similar tweet filtering work [27,28,52]. To determine the best parameterisation for each classifier, a grid-search method was adopted to train and evaluate the models over a range of parameters. All models used a preprocessing step that applied term frequency inverse document frequency (TF-IDF) to convert tweet text into a numerical input vector. TF-IDF highlights terms that are locally frequent but globally infrequent, increasing differentiation between vectors to improve classification.

The outcome of the three grid-searches is shown in Table 2 with the final model parameters alongside the different performance metrics. For assessing the grid-search, a macro F1-Score was used as the performance metric due to the dataset being unbalanced, with 1952 relevant and 605 irrelevant filtered tweets (with a relevance of 73%).

Note that Logistic Regression and the Support Vector Machine have the first and second highest precision respectively, and Multinomial Naïve Bayes and Logistic Regression have first and second highest recall, respectively. As each of the three classifiers performed better in certain metrics than at least one other model, a multi-model ensemble classifier was set up, where if two of the three classifiers agreed then that decision would be taken. Apart from the recall of the Multinomial Naive Bayes, this performed better than the individual classifiers. Importantly, this classifier performed significantly better than a model which always selected the most prevalent class, which would have achieved a 73% accuracy. After filtering the data with the classifier, 518,167 relevant tweets were retained (27% of Dataset 1’s original volume). A manual check showed a classification accuracy of 97% from 200 randomly sampled tweets.

##### Application to Dataset 2

After applying the filtering steps outlined above to Dataset 1 (June–August 2018), the same process was repeated for Dataset 2 (December 2018–February 2019). The preprocessing used the same parameters and filters as for Dataset 1, with manual checks after each step confirming that there were no irregularities found exclusively in the new data. Like Dataset 1, the ensemble classifier performed better than any individual model, further justifying its use, with a sample of 200 tweets showing a final Dataset 2 relevance of 86%. A summary of the tweet volume after each stage is shown in Figure 1b, with the final filtered tweet count of 140,361 (19% of the Dataset 2’s original volume).

##### Location Inference

As only around 1.2% of filtered collected tweets are geotagged, additional methods are required to infer the location. The location inference technique used was the same as that of Arthur et al. [28] (adapted from that of Schulz et al. [53]), and can be found at https://github.com/seda-lab/social_sensing (accessed on 20 August 2020). The technique searches multiple ‘indicators’ in the tweet metadata, most importantly: the tweet text, and the user’s defined location for place names. Upon searching the GADM [54], DBpedia [55] and GeoNames [56] gazetteers, a set of coordinates or bounding boxes containing locations found in the tweet objects are returned. Finally, to infer the most probable location, areas of overlap between the locations are detected before a final coordinate or bounding box is returned. 82% of the Dataset 1 filtered tweets and 80% of the Dataset 2 filtered tweets were successfully geolocated, with a final reduction to 423,932 and 111,785 tweets, respectively. Schulz et al. [53] found that this approach has a median accuracy of below 30 km, with 22% of tweets being located within 1 km.

#### 2.2.2. Temperature Data

The MIDAS temperature data required no preprocessing. For the NOAA data, missing entries marked as 9999 ${}^{\circ }$F were removed. Due to the volume of entries (UK: 33,000, US: 98,000, Australia: 31,000), any remaining outliers are unlikely to noticeably impact analysis.

Unlike the evenly distributed UK weather stations, stations in the US and Australia are mostly distributed on the east coasts of the countries. Therefore, the calculated national average temperatures across weather stations will not provide the true mean temperatures across space. For this study, the average across stations still provides a suitable approximation and has been used in similar work (e.g., Grasso et al. [57]).

#### 2.2.3. Sentiment Analysis

Sentiment analysis is a field of natural language processing that aims to extract the attitude conveyed in a body of text. For this project, sentiment analysis was performed using the Python library vaderSentiment (Vader) [58]. Upon applying Vader to text, a compound metric between −1 (extremely negative) and 1 (extremely positive) is returned. Vader was chosen for this work due to its ability to comprehend slang/emojis and effectiveness on short texts (as shown in Table 3).

## 3. Results

### 3.1. Country Scale Analysis

**Figure 2:**
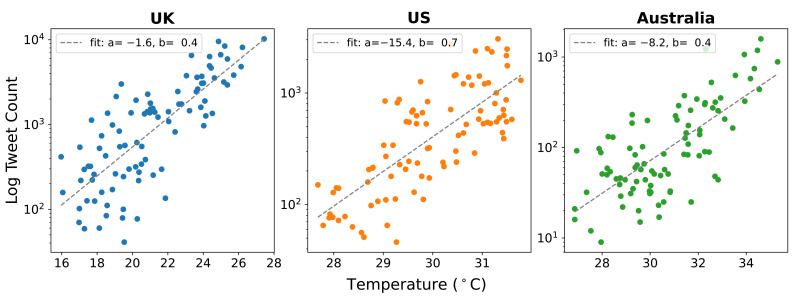
Scatter plot comparing the log daily tweet count to the average daily maximum temperature (ADMT) for each country. The line of best fit is modelled by log(y) = a + bx.

Figure 2 shows a collection of scatter plots between the average daily maximum temperature (ADMT) in each country, and the logarithmic-scaled daily tweet count. All three plots demonstrate a strong positive correlation such that as the temperature increases the number of tweets discussing heatwaves increases exponentially (UK Pearson’s Coeff: 0.7836, *p* < 0.0001, US Pearson’s Coeff: 0.7556, *p* < 0.0001, Australia Pearson’s Coeff: 0.7218, *p* < 0.0001). This relationship is modelled by the equation log(y) = a + bx and the best fit is shown in Figure 2, with parameters in the upper-left of each subplot. The range of temperature variation in the UK is greater than both the US and Australia, but UK temperatures are typically lower.

**Figure 3:**
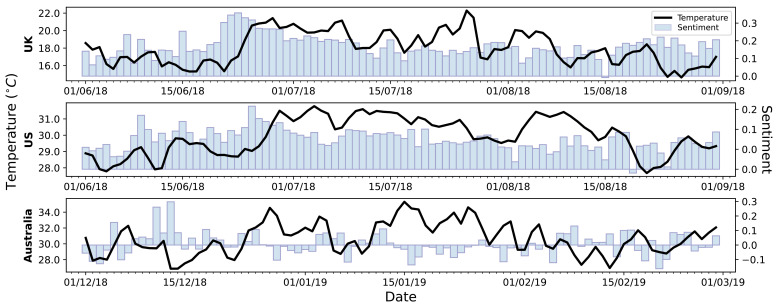
Bar chart of sentiment change throughout the summer months from tweets within each country, with ADMT plotted in black.

Figure 3 shows how the average daily sentiment polarity of heatwave tweets fluctuates alongside ADMT. For the UK subplot, there is a sentiment increase in the days leading up to the first substantial temperature peak on 28th June. The sentiment then gradually decreases as the summer continues, with tweets during the period’s largest temperature peak (26th July) being noticeably more negative than the tweets around the first temperature peak. The US subplot follows a similar trend to the UK, with a sentiment increase leading up to the first temperature peak, before gradually decreasing throughout the summer. The sentiment within Australian heatwave tweets fluctuates throughout the summer, with a higher level of negativity than the other countries. During this period in Australia there is a significant but weak negative Pearson’s correlation coefficient between temperature and sentiment (Pearson’s Coeff: −0.2615, *p* = 0.0132), implying that as temperature increases, sentiment decreases. Note that a statistically significant correlation between these variables is not seen within the UK and US results (Pearson’s Coeff: 0.1588, *p* = 0.1306 and Pearson’s Coeff: 0.1651, *p*= 0.1158, respectively).

**Figure 4:**
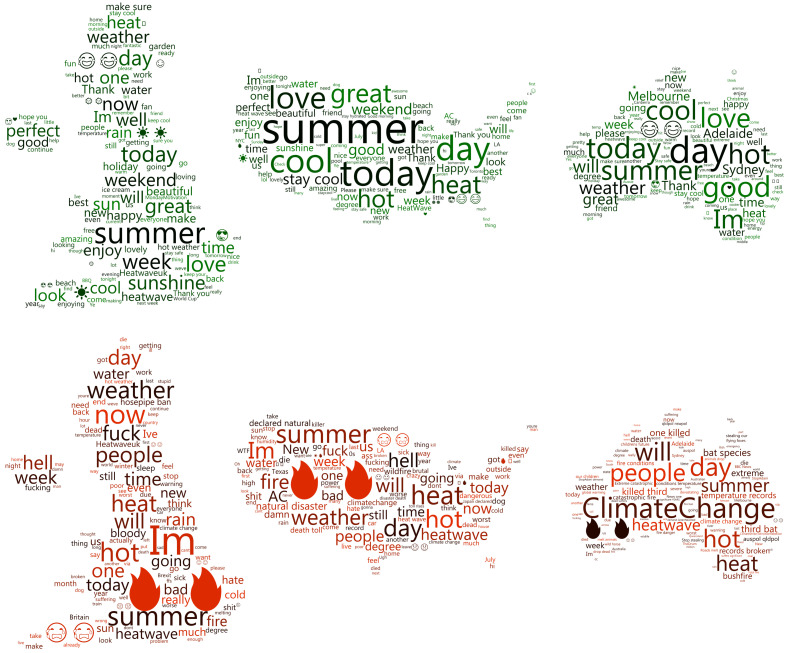
Word clouds showing the language used in the extremely positive (**top**) and negative (**bottom**) tweets in each country during the summer months.

As heatwaves are generally defined according to local rather than national conditions, the following analysis presents the language used to discuss heatwaves during the summer months, rather than explicitly during a heatwave. To observe the topics and language most passionately discussed within the individual countries, Figure 4 shows word clouds using the tweets with sentiment above/below a ±0.75 threshold, that is, grouping the extremely positive and extremely negative messages in each country. Before the word clouds were generated, the tweets were processed to remove mentions, punctuation, and URLs. In addition to this, a list of words have been excluded from the refined tweets, including common words (‘and’, ‘the’, ‘a’) as well as terms such as ‘heatwave’ and country names. Pronouns have not been removed from the word clouds as they can offer insight into the overall theme or subject of the tweets.

The top row of Figure 4 shows the commonly used language in the positive heatwave tweets within the three countries. One of the most common words for the US and Australia is ‘cool’ with both nations having fewer ‘hot’ uses than ‘cool’ uses. Whilst all three plots are similar, the UK appears to have more discussion regarding potential heatwave activities, such as ‘ice-cream’, ‘world cup’, ‘beach’, ‘sunshine’ and ‘garden’.

The bottom row of Figure 4 shows the language within the thresholded negative sentiment heatwave tweets. The Australian plot seems to focus on national issues, for instance ‘Climate Change’, ‘temperature records’ and ‘children’s futures’, as well as concern for the natural world, including ‘bat species’, ‘fish’ and ‘bushfire’. The US plot is similar with ‘natural disaster’, ‘climate change’ and ‘fire’; however, more emotion is present with ‘crazy’, ‘fuck’ and ‘bad’, alongside topics of potential inconvenience such as ‘AC’. Finally, the UK word cloud is centred around personal discomfort and inconveniences, with terms ‘hell’, ‘hosepipe ban’, ‘fuck’ and ‘sleep’.

### 3.2. City Scale Trends

**Figure 5:**
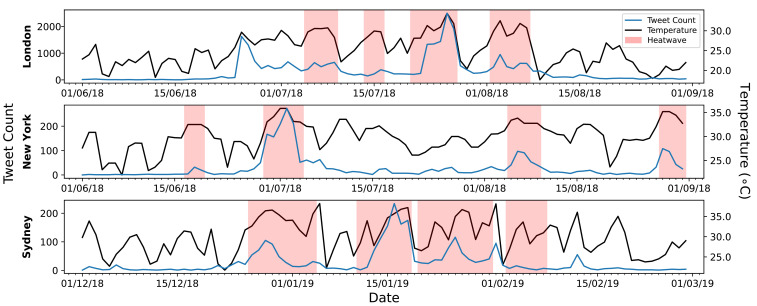
Daily tweet count in the investigated cities, overlaid with the government defined heatwave days and the average daily maximum temperature (ADMT).

Next, an investigation into the trends in the three largest cities in the above countries was conducted. There were approximately 30,000 tweets from London (18% of the UK total), approximately 2500 tweets from New York (5% of the US total), and approximately 2500 tweets from Sydney (14% of the Australian total), with the cities having 8, 2 and 14 weather stations, respectively. Using the local heatwave definitions defined earlier (London: 3 days over 28 ${}^{\circ }$C, New York: 3 days over 90 ${}^{\circ }$F (32 ${}^{\circ }$C), Sydney: EHF definition), Figure 5 shows a comparison of the tweet count to defined heatwave days in each city. The early peak for London on 25th July was the first temperature spike of the year, with the Met Office issuing a Level 3 heat-health watch alert for a large part of England a day earlier [59]. The New York heatwave definition strongly aligns with the tweet influxes over the summer, with few tweets about heatwaves between events. Despite containing 90% fewer tweets than London, both cities have similarly shaped main heatwave events. Additionally, the rate of increase in these cities is similar, with sudden tweet spikes on the first day of each event. The Sydney heatwave days cover most of the significant temperature spikes, with tweet count increasing during most temperature increases.

**Figure 6:**
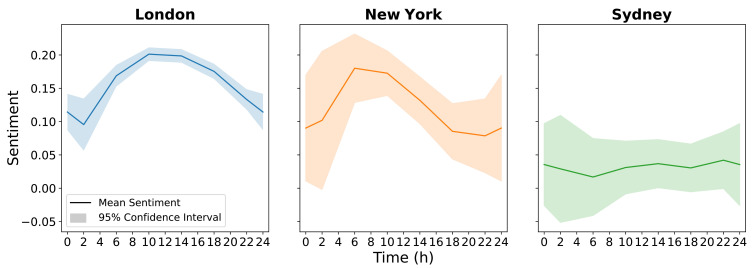
Average sentiment over the heatwave days for each city with a 95% confidence interval.

Having observed a relationship between tweets on a city scale and heatwave events, we next examined public attitudes in each city when discussing heatwaves. For this analysis, the tweets during each city’s summer were resampled into four-hour windows, before the average sentiment for the tweets in each window was calculated to show how sentiment changed over a 24-h period. The 4-h window size provided a suitable balance between volume (i.e., high tweet counts in each 4-h period allowing accurate averages to be calculated) and resolution (enabling trends over 24 h to be observed). Figure 6 shows each city’s sentiment fluctuation, with the plots having been adjusted to accommodate for time zones. London’s curve is parabolic, showing a clear rise in sentiment, peaking between 10:00 and 14:00, before a decrease into the evening. The 95% confidence interval (CI) is consistent, with a slightly wider range in the early hours reflecting lower tweet counts while many people are asleep. The confidence interval in New York is larger throughout and appears to follow a similar trend to London, with the exception that the sentiment peak is seen earlier in the day. The sentiment range for New York is also similar to that for London, albeit with less confidence due to the lower data volumes. Sydney has a similar confidence interval to New York, and there is no evidence for a trend in sentiment throughout the day. The sentiment in Sydney is consistently more negative than the sentiment from both London and New York.

## 4. Discussion

This investigation aimed to identify the utility of social sensing using Twitter data to detect the social impacts of heatwaves in the UK, US and Australia. Based on the analysis above, there is good evidence that social media (Twitter) user populations respond to heatwaves through the volume and sentiment of their posts, and trends regarding heatwaves can be identified at both national and city scale. These trends are consistent with findings from other traditional survey and sensor methodologies, but can be generated quickly at minimal cost, highlighting the potential value of social sensing for heatwave preparedness and management.

Despite most tweets being produced in the UK, the UK heatwave was not more severe or intense than the US and Australian heatwaves, with the UK having the mildest average temperature range. Aside from the varying popularity of Twitter in each country, another potential explanation for the variation in Twitter response is that the frequency of tweets in an area during a heatwave is based on how anomalous the heat condition is to the local climate, rather than the actual temperature. This is backed up through Table 1, with the UK having the largest temperature difference between average conditions and conditions in the researched period. This supports findings by Giuffrida et al. [60] and Nairn and Fawcett [18]. It was found that all three counties showed an exponential increase in Twitter activity as the temperature increased, though with different growth rates. Whilst this study has not determined the cause of this strong relationship, a potential explanation is social reinforcement. For example, as people begin to tweet about an event, others see these tweets and follow up with tweets/replies/retweets. These social interactions increase the event’s exposure (particularly retweets due to their exponential growth [61]), which can result in trending on Twitter, furthering the social reach and coverage. Another speculative explanation is through thermal discomfort, with Pyrgou and Santamouris [62] showing an exponential increase in mortality rates during heatwaves, which is potentially reflected through Twitter. Future work in this area might help to disambiguate these possible explanations.

The UK and US demonstrated similar levels of positivity throughout the heatwaves, whereas Australia was noticeably more negative in its response. Despite the weak correlation, Australia demonstrated a statistically significant sentiment increase as temperature decreased. Whilst the positive language used in each country was similar, the UK had more of an emphasis on the activities enabled by the heat, whereas the US and Australia had language focussed on the relief from the heat. The negative language used in the UK was more focussed on short-term human impacts of heatwaves, the Australian tweets were more concerned with large scale impacts, with a clear emphasis on the environmental consequences, and the US was somewhat concerned with both. The use of the pronoun ‘I’m’ within the word clouds reinforces this, with it being the most common word in the UK data, but not very frequent in Australia. These results corroborate the findings of Bruine de Bruin et al. [12], Howe et al. [15] and Akompab et al. [19], further highlighting the underestimation of heatwave impacts in the UK and US, which as stated by Howe et al. [15], increases the health risk for individuals.

The difference in response to heatwaves within London, New York and Sydney was analysed, with visual correlations between tweet count and each city’s governmentally defined heatwave days. Within London, despite the temperature during the first tweet spike not satisfying the formal Met Office heatwave definition, there was still a large amount of London heatwave chatter. This is potentially due to shock or surprise at the temperature spike and the lack of acclimatisation, further suggesting that tweets are a result of the sudden temperature change and current discomfort level, rather than the actual temperature. This is reinforced by the final “official” heatwave between 2nd–7th August in London receiving fewer tweets than the first non-official heatwave spike. The similar rapid rate of increase in New York and London tweet counts during heatwaves implies that the heat is less expected and that the shock at the beginning of the event is tweet-worthy. Overall, it is interesting to note that spikes of attention and response to heat do not always correspond to heatwave events as defined by local meteorological agencies. This mismatch between official recognition and social response suggests that definitions based solely on temperature or weather conditions may not always reflect human perceptions of heat.

The city-wide sentiment over a 24-h period showed that both London and New York had sentiment peaks during the early part of each day and dips during the night time, whereas Sydney showed reasonably consistent low sentiment at all times. A speculative explanation for this trend in London and New York is that heat during the nighttime causes negativity from the discomfort of being unable to sleep, while the morning/midday peak is in pleasurable anticipation of a warm day. In both Australia and Sydney there is generalised negativity about heat, consistent with heat and drought being seen as a hazard in the hotter/drier climate [19]. However, more data is required to validate these explanations. This section has offered an increased level of understanding into the time of day city populations are most negative about heat, as well as the stage in the heatwave period that elicits the greatest social media response. Being aware of these two factors could contribute to the decision making of local governments seeking to mitigate the possible effects on the population.

## 5. Limitations and Future Scope

There were limitations within this study, with the opportunity for future research highlighted. One such limitation is the usage and demographics of Twitter. In the US, UK and Australia, 22%, 23% and 21% of the population use Twitter, respectively, with 87.3% of the Twitter population being under 50 [36,37,38,63]. As heatwaves are a greater risk to the elderly and vulnerable, it is worth being mindful that these two demographics were likely underrepresented in the results. By identifying demographics on Twitter using a similar technique to Vijayaraghavan et al. [64], the heatwave signal produced by vulnerable demographics could be enhanced, potentially reducing demographic bias in the results. Future investigation into the risks for different demographics would be valuable. However, the concern about demographic representativeness depends on the purpose of the analysis. If the aim is simply to detect heatwaves and locate them in time and space, it is unlikely to matter which parts of a population are writing the tweets; one person’s observation of heat may be as good as another’s. However, if the aim is to study social impacts and population vulnerability to heatwaves, it becomes much more important to account for demographics and ensure that all parts of society are served.

The language used on Twitter is notoriously sarcastic and hyperbolic, causing potential linguistic limitations within this project [65]. For instance, a tweet about heatwaves may be classified incorrectly by the sentiment analysis technique due to its lack of sarcasm comprehension. Anderson and Huntington [66] found that sarcasm levels in tweets regarding climate change were low, so if this reflects a general lack of sarcasm around weather or other natural phenomena, then sarcasm may be less of a factor in the current analysis of heatwave tweets. One limitation in the current study arises from the search term used to collect Twitter data; Twitter users tend to use colloquial terms, so tweets that use the specific word ‘heatwave’ may not be a representative sample of all heatwave-related content. For future work, exploring impact terms, such as ‘sunburn’ and ‘dehydration’, alongside conversational terms for describing heatwaves such as ‘heat’ and ‘hot’ is recommended, though this will make relevance filtering much more challenging.

## 6. Conclusions

As the frequency, duration and intensity of heatwaves continue to grow, the necessity of understanding and managing the impacts of extreme heat has never been greater. Social sensing can be a valuable technology for detecting heatwaves and the varying attitudes concerning them. On a national scale, there is a statistically significant exponential correlation between temperature and social media activity. Between countries, social sensing detects temporal fluctuations in attitude as well as varying heatwave perceptions. On a city scale, the social response closely aligns with governmentally defined heatwave days, with predictable sentiment changes over 24 h. This demonstrates the feasibility of a heatwave response framework informed by social sensing, similar to the Met Office Twitter-based flood impact detection system implemented by Arthur et al. [28]. These results provide insight into the social impacts of heatwaves, including how and when a population’s wellbeing is most affected and how behavioural changes are exacerbated by heat. This deeper understanding of heatwaves can complement additional sources such as emergency response data and health records, bettering the management and response to heatwaves, and potentially improving the lives of many. 

## Figures and Tables

**Table 1:** Summary of researched summer conditions compared to previous conditions [11].

Country	Average Summer Temperature (${}^{\circ }$C)	Investigated Summer Period	Average Temperature in Investigated Period (${}^{\circ }$C)
UK	14.8	July–September 2018	17.5
US	21.8	July–September 2018	24.0
Australia	22.5	December–February 2018–2019	24.1

**Table 2:** Final model parameters and performance metrics trained on Dataset 1.

Classifier	Model Parameters		Accuracy	Precision	Recall	F1 Score
Multinomial Naïve Bayes	Alpha: Fit Prior:	0.5 False	0.886	0.747	0.781	0.764
Logistic Regression	C: Penalty: Solver:	0.5 None LBFGS	0.895	0.800	0.742	0.770
Support Vector Machine	C: Kernel:	2 Linear	0.889	0.782	0.735	0.758
Multi-Model Ensemble	Assign Majority Classification		0.908	0.833	0.762	0.796

**Table 3:** Sentiment classified fictitious tweets using similar language to the dataset.

Text	Sentiment Score
Can’t wait for the heatwave next week 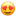	0.5692
It is far too hot! I H8 the heatwave	−0.6932
My dog really doesnt like the heatwave. I love it haha	0.7014
Free Ice Cream at work? Yes please 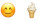 #heatwave	0.8689
I am on holiday and there is a UK heatwave!? BLOODY SELFISH!	−0.7469

## Data Availability

The Twitter data used in this word was collected using the official API (https://developer.twitter.com/en/docs/twitter-api (accessed on 20 August 2020)). The temperature data was collected from the NOAA GSOD dataset (https://www.ncei.noaa.gov/access/search/data-search/global-summary-of-the-day (accessed on 15 August 2020)) and the Met Office MIDAS dataset (https://catalogue.ceda.ac.uk/uuid/220a65615218d5c9cc9e4785a3234bd0 (accessed on 15 August 2020)).

## Appendix A

**Table A1:** Common terms in usernames excluded from analysis. This list was derived through manual observation of active weather stations or news accounts. These accounts did not provide a social perspective to heatwaves and were removed.

Term	Username Frequency
news	21,158
eco	4509
radio	3451
weather	3398
farm	2994
daily	2755
water	2623
climate	1865
heat	1561
press	1434
post	1335
times	1302
bbc	1128
drought	1072
storm	823
story	468
network	404
trending	378
gazette	376
independent	326
stories	309
thesun	232

**Table A2:** Through inspecting manually classified irrelevant tweets, there were five common irrelevant tweet topics. Key terms within each topic were compiled into a list and if a tweet contained any of these terms, it was excluded.

Topic	Terms	Tweets
Korean Pop Music	k-pop, kpop, korean pop, blackpink, bts, jungkook, suga, j-hope, jimin, bts, exo, xiumin, suho, baekhyun, chanyeol, sehun, kyungsoo	16,961
Western Pop Music	love drought, album, beyonce, pop drought	7240
Sports	playoff, nfl, mlb, wwe, team	12,000
Sexual	pussy, dick	2385
Other	dorito, retweet, rt	6581

## References

[1] Mukherjee S., Mishra A.K. (2021). Increase in Compound Drought and Heatwaves in a Warming World. Geophys. Res. Lett..

[2] NOAA What Is a Heat Wave?|NOAA SciJinks—All About Weather. https://scijinks.gov/heat/.

[3] Robine J.M., Cheung S.L.K., Le Roy S., Van Oyen H., Griffiths C., Michel J.P., Herrmann F.R. (2008). Death Toll Exceeded 70,000 in Europe during the Summer of 2003. Comptes Rendus Biol..

[4] Gilbert N. (2010). Russia counts environmental cost of wildfires. Nature.

[5] Kjellstrom T., Maître N., Saget C., Otto M., Karimova T. (2019). Working on a Warmer Planet: The Effect of Heat Stress on Productivity and Decent Work Report.

[6] Liu X., Liu H., Fan H., Liu Y., Ding G. (2019). Influence of Heat Waves on Daily Hospital Visits for Mental Illness in Jinan, China—A Case-Crossover Study. Int. J. Environ. Res. Public Health.

[7] Gao J., Cheng Q., Duan J., Xu Z., Bai L., Zhang Y., Zhang H., Wang S., Zhang Z., Su H. (2019). Ambient Temperature, Sunlight Duration, and Suicide: A Systematic Review and Meta-Analysis. Sci. Total Environ..

[8] McCarthy M., Armstrong L., Armstrong N. (2019). A New Heatwave Definition for the UK. Weather.

[9] Xu Z., FitzGerald G., Guo Y., Jalaludin B., Tong S. (2016). Impact of Heatwave on Mortality under Different Heatwave Definitions: A Systematic Review and Meta-Analysis. Environ. Int..

[10] Guo Y., Gasparrini A., Armstrong B.G., Tawatsupa B., Tobias A., Lavigne E., Coelho M.D., Pan X., Kim H., Hashizume M. (2017). Heat Wave and Mortality: A Multicountry, Multicommunity Study. Environ. Health Perspect..

[11] Weatherbase (2020). Weather Averages—All Countries (Weatherbase). https://www.weatherbase.com/weather/countryall.php3.

[12] Bruine de Bruin W., Lefevre C.E., Taylor A.L., Dessai S., Fischhoff B., Kovats S. (2016). Promoting Protection against a Threat That Evokes Positive Affect: The Case of Heat Waves in the United Kingdom. J. Exp. Psychol. Appl..

[13] Ortiz L.E., Gonzalez J.E., Wu W., Schoonen M., Tongue J., Bornstein R. (2018). New York City Impacts on a Regional Heat Wave. J. Appl. Meteorol. Climatol..

[14] NOAA, US Department of Commerce (2019). Weather Related Fatality and Injury Statistics.

[15] Howe P.D., Marlon J.R., Wang X., Leiserowitz A. (2019). Public Perceptions of the Health Risks of Extreme Heat across US States, Counties, and Neighborhoods. Proc. Natl. Acad. Sci. USA.

[16] Abrahamson V., Wolf J., Lorenzoni I., Fenn B., Kovats S., Wilkinson P., Adger W.N., Raine R. (2009). Perceptions of Heatwave Risks to Health: Interview-Based Study of Older People in London and Norwich, UK. J. Public Health.

[17] BOM (2019). Tracking Australia’s Climate through 2019.

[18] Nairn J., Fawcett R. (2014). The Excess Heat Factor: A Metric for Heatwave Intensity and Its Use in Classifying Heatwave Severity. Int. J. Environ. Res. Public Health.

[19] Akompab D.A., Bi P., Williams S., Grant J., Walker I.A., Augoustinos M. (2013). Awareness of and Attitudes towards Heat Waves within the Context of Climate Change among a Cohort of Residents in Adelaide, Australia. Int. J. Environ. Res. Public Health.

[20] Yang J., Hu L., Wang C. (2019). Population Dynamics Modify Urban Residents’ Exposure to Extreme Temperatures across the United States. Sci. Adv..

[21] Li D., Bou-Zeid E. (2013). Synergistic Interactions between Urban Heat Islands and Heat Waves: The Impact in Cities Is Larger than the Sum of Its Parts. J. Appl. Meteorol. Climatol..

[22] Gao Z., Hou Y., Chen W. (2019). Enhanced Sensitivity of the Urban Heat Island Effect to Summer Temperatures Induced by Urban Expansion. Environ. Res. Lett..

[23] Dong J., Peng J., He X., Corcoran J., Qiu S., Wang X. (2020). Heatwave-Induced Human Health Risk Assessment in Megacities Based on Heat Stress-Social Vulnerability-Human Exposure Framework. Landsc. Urban Plan..

[24] Robbins J.C., Titley H.A. (2018). Evaluating High-Impact Precipitation Forecasts from the Met Office Global Hazard Map (GHM) Using a Global Impact Database. Meteorol. Appl..

[25] Monti C., Rozza A., Zappella G., Zignani M., Arvidsson A., Colleoni E. (2013). Modelling political disaffection from Twitter data. Proceedings of the Second International Workshop on Issues of Sentiment Discovery and Opinion Mining.

[26] Williams M.L., Burnap P., Sloan L. (2016). Crime Sensing with Big Data: The Affordances and Limitations of Using Open Source Communications to Estimate Crime Patterns. Br. J. Criminol..

[27] Sakaki T., Okazaki M., Matsuo Y. (2010). Earthquake shakes Twitter users: Real-time event detection by social sensors. Proceedings of the 19th International Conference on World Wide Web.

[28] Arthur R., Boulton C.A., Shotton H., Williams H.T.P. (2018). Social Sensing of Floods in the UK. PLoS ONE.

[29] Spruce M., Arthur R., Williams H.T.P. (2020). Using Social Media to Measure Impacts of Named Storm Events in the United Kingdom and Ireland. Meteorol. Appl..

[30] Boulton C., Shotton H., Williams H. Using Social Media to Detect and Locate Wildfires. Proceedings of the Tenth International AAAI Conference on Web and Social Media.

[31] Cepni K., Ozger M., Akan O.B. (2018). Event Estimation Accuracy of Social Sensing With Facebook for Social Internet of Vehicles. IEEE Internet Things J..

[32] Baylis P., Obradovich N., Kryvasheyeu Y., Chen H., Coviello L., Moro E., Cebrian M., Fowler J. (2017). Weather Impacts Expressed Sentiment. PLoS ONE.

[33] Jayarajah K., Misra A. Can Instagram Posts Help Characterize Urban Micro-Events?. Proceedings of the 2016 19th International Conference on Information Fusion (FUSION).

[34] Silva T., Vaz de Melo P., Salles J., Loureiro A. A Picture of Instagram is Worth More Than a Thousand Words: Workload Characterization and Application. Proceedings of the 2013 IEEE International Conference on Distributed Computing in Sensor Systems, DCoSS.

[35] Wu D., Cui Y. (2018). Disaster early warning and damage assessment analysis using social media data and geo-location information. Decis. Support Syst..

[36] VividSocial (2020). Social Media Statistics Australia—January 2020.

[37] Statista (2020). Twitter: Most Users by Country.

[38] Wojcik S. (2019). Sizing Up Twitter Users. https://www.pewresearch.org/internet/2019/04/24/sizing-up-twitter-users/.

[39] Twitter Twitter API Documentation. https://developer.twitter.com/en/docs/twitter-api.

[40] Mayors C. (2018). City Mayors: Largest Cities in the World by Population (1 to 125). http://www.citymayors.com/statistics/largest-cities-population-125.html.

[41] McCarthy M., Christidis N., Dunstone N., Fereday D., Kay G., Klein-Tank A., Lowe J., Petch J., Scaife A., Stott P. (2019). Drivers of the UK Summer Heatwave of 2018. Weather.

[42] NOAA (2018). Assessing the U.S. Climate in August 2018.

[43] BOM (2019). Special Climate Statements.

[44] Twitter (2013). New Tweets Per Second Record, and How.

[45] Crockford D. (2006). JSON: The Fat-Free Alternative to XML. https://www.json.org/fatfree.html.

[46] Twitter Developers (2020). Developer Agreement and Policy.

[47] Arthur R., Williams H.T.P. (2019). Scaling Laws in Geo-Located Twitter Data. PLoS ONE.

[48] Met Office (2019). Met Office MIDAS Open: UK Land Surface Stations Data (1853-Current).

[49] National Climatic Data Center, NESDIS, NOAA, U.S. Department of Commerce Dataset Overview|National Centers for Environmental Information (NCEI): Silver Spring, MD, USA. https://www.ncei.noaa.gov/access/metadata/landing-page/bin/iso?id=gov.noaa.ncdc:C00516.

[50] Kwak H., Lee C., Park H., Moon S. (2010). What Is Twitter, a Social Network or a News Media?. Proceedings of the 19th International Conference on World Wide Web.

[51] Danilak M.M. (2020). Langdetect: Language Detection Library Ported from Google’s Language-Detection. https://github.com/Mimino666/langdetect.

[52] Indra S.T., Wikarsa L., Turang R. Using Logistic Regression Method to Classify Tweets into the Selected Topics. Proceedings of the 2016 International Conference on Advanced Computer Science and Information Systems (ICACSIS).

[53] Schulz A., Hadjakos A., Paulheim H., Nachtwey J., Mühlhäuser M. A Multi-Indicator Approach for Geolocalization of Tweets. Proceedings of the 7th International Conference on Weblogs and Social Media, ICWSM 2013.

[54] GLOBE (2012). Global Administrative Areas.

[55] Auer S., Bizer C., Kobilarov G., Lehmann J., Cyganiak R., Ives Z., Aberer K., Choi K.S., Noy N., Allemang D., Lee K.I., Nixon L., Golbeck J., Mika P., Maynard D., Mizoguchi R. (2007). DBpedia: A Nucleus for a Web of Open Data. The Semantic Web.

[56] GeoNames The GeoNames Geographical Database Covers All Countries and Contains Over Eleven Million Placenames That Are Available for Download Free of Charge. https://www.geonames.org/.

[57] Grasso V., Crisci A., Morabito M., Nesi P., Pantaleo G. (2017). Public Crowdsensing of Heat Waves by Social Media Data. Adv. Sci. Res..

[58] Hutto C., Gilbert E. VADER: A Parsimonious Rule-Based Model for Sentiment Analysis of Social Media Text. Proceedings of the 8th International Conference on Weblogs and Social Media, ICWSM 2014.

[59] Met Office (2018). Heatwave Continues with Temperatures into the Mid 30 s Celsius.

[60] Giuffrida L., Lokys H., Klemm O. (2020). Assessing the Effect of Weather on Human Outdoor Perception Using Twitter. Int. J. Biometeorol..

[61] Encore (2015). Favorites vs. Retweets (And Why One Is More Important Than the Other).

[62] Pyrgou A., Santamouris M. (2018). Increasing Probability of Heat-Related Mortality in a Mediterranean City Due to Urban Warming. Int. J. Environ. Res. Public Health.

[63] Statista (2020). Global Twitter User Age Distribution 2020.

[64] Vijayaraghavan P., Vosoughi S., Roy D. Twitter Demographic Classification Using Deep Multi-Modal Multi-Task Learning. Proceedings of the 55th Annual Meeting of the Association for Computational Linguistics (Volume 2: Short Papers), Association for Computational Linguistics.

[65] Bouazizi M., Ohtsuki T. Sarcasm Detection in Twitter: “All Your Products Are Incredibly Amazing!!!”—Are They Really?. Proceedings of the 2015 IEEE Global Communications Conference (GLOBECOM).

[66] Anderson A.A., Huntington H.E. (2017). Social Media, Science, and Attack Discourse: How Twitter Discussions of Climate Change Use Sarcasm and Incivility. Sci. Commun..

